# Changes in cardiovascular parameters in rats exposed to chronic widespread mechanical allodynia induced by hind limb cast immobilization

**DOI:** 10.1371/journal.pone.0245544

**Published:** 2021-01-19

**Authors:** Takahiko Yoshimoto, Hiroki Sakurai, Yusuke Ohmichi, Mika Ohmichi, Atsuko Morimoto, Takahiro Ushida, Jun Sato

**Affiliations:** 1 Department of Hygiene, Public Health and Preventive Medicine, Showa University School of Medicine, Shinagawa, Tokyo, Japan; 2 Multidisciplinary Pain Center, Aichi Medical University, Nagakute, Aichi, Japan; 3 Faculty of Health Sciences, Tokoha University, Hamamatsu, Japan; 4 Department of Anatomy, Aichi Medical University, Nagakute, Aichi, Japan; 5 Department of Physical Therapy, College of Life and Health Sciences, Chubu University, Kasugai, Japan; University of Pittsburgh, UNITED STATES

## Abstract

To elucidate the relationship between chronic pain conditions with cast immobilization and autonomic function, we investigated the functional changes of the autonomic nervous system in conscious rats with chronic post-cast pain (CPCP) induced by a two-week cast immobilization of one hind limb. We telemetrically examined the time courses of systolic arterial blood pressure (SBP), heart rate (HR), and the middle-frequency (MF) component obtained from the power spectral analysis of SBP variability as a vasomotor sympathetic index. We also investigated the baroreflex sensitivity to phentolamine, an α-adrenoceptor antagonist, and the SBP and HR responses to a low ambient temperature (LT; 9.0 ± 0.2°C) exposure, a sympathetic stimulant. Rats exposed to cast immobilization exhibited mechanical allodynia lasting for at least 10 weeks after cast removal in the calf area (skin and muscle) of the bilateral hind limbs. Under resting conditions, the SBP, HR, and MF components were significantly increased during cast immobilization (all *p* < 0.001). Following cast removal, these parameters gradually decreased and within 1 week reached lower than baseline levels, lasting for over 10 weeks. Phentolamine administration (10 mg/kg, intraperitoneally) significantly decreased the SBP before and during cast immobilization (before, *p* < 0.001; during, *p* = 0.001) but did not lower the SBP after cast removal. The baroreflex gain after phentolamine administration, calculated as the HR increase divided by the SBP reduction, was significantly increased after cast removal (*p* = 0.002). The SBP increase on LT exposure was significantly greater after cast removal than that before cast immobilization, suggesting hypersensitivity to sympathetic neurotransmitters. These results revealed that, in the CPCP model, sympathetic activation was augmented during cast immobilization, which then decreased after cast removal and remained below normal levels with persisting pain behaviors. Additionally, the responsiveness of the autonomic nervous system was impaired in the CPCP model.

## Introduction

Chronic pain is often accompanied by autonomic disorders, as well as spontaneous pain, hyperalgesia, and allodynia [1–5]. Complex regional pain syndrome (CRPS) is characterized by changes in skin temperature and skin blood flow in the affected area along with chronic widespread pain. It has been suggested that the development and maintenance of this pathological condition are closely related to the dysfunction of the autonomic nervous system, especially the excitation of the sympathetic nervous system (SNS). A previous study in patients with CRPS reported vascular dysfunctions, such as enhanced vasoconstrictive responses to noradrenaline in the affected limb [6]. In animal models of chronic pain, abnormal changes in the peripheral vascular functions have also been reported [7–9], suggesting that SNS excitation is also involved in neuropathic pain conditions. However, it has been shown that sympathetic activity in the affected limb is not increased in CRPS [10–12]. Previous studies in patients with CRPS also showed that the venous level of the sympathetic neurotransmitter noradrenaline was lower in the affected limb than in the unaffected limb [13–16]. These findings indicate that reduced vascular sympathetic activity in the affected extremity leads to a warm extremity during the acute phase of CRPS. Decreased levels of neurotransmitters, however, could subsequently cause an augmented responsiveness of α-adrenoceptors on blood vessels [17, 18], resulting in a cold extremity in the chronic stage of CRPS. This is demonstrative that the activity of sympathetic neurons is normal or decreased but never increased in chronic pain conditions.

In addition to studies focusing on regional sympathetic dysfunction in the affected extremity, systemic sympathetic dysfunction has been reported in rats [19] or humans [20–22]. Recently, some studies have investigated the cardiovascular function in animal models of chronic pain [7, 23–25]. Although it is known that SNS dysfunction in chronic pain varies with the pathological state, few studies have followed the time course of the function of the autonomic nervous system. In rats with chronic constriction injury (CCI) of the sciatic nerve in an animal model of chronic pain, arterial blood pressure (BP) and heart rate (HR) were significantly increased up to 7 days following CCI surgery and then declined to baseline levels or below [7]. The same authors also observed that plasma noradrenaline levels temporally increased 3–6 days after surgery and then returned to the pre-surgery level. These findings indicate that although the cardiac SNS activity in the chronic pain model is increased during early postoperative periods, these effects do not last long and reverse subsequently. These results also imply that the activity of the SNS changes in chronic pain conditions along with the pathological state.

It has been reported that the hind limb immobilization of rats, with a cast for 2 weeks, induces local inflammation in the immobilized hind limb after cast removal and causes persistent mechanical allodynia in both hind limbs [26, 27]. We consider that these changes are similar to the pathological condition of widespread pain observed after immobilization in humans. Although it has been reported in humans that immobilization is accompanied by the dysfunction of the autonomic nervous system [28, 29], the mechanisms are not yet fully understood. Therefore, the main purpose of this study was to investigate the functional changes of the autonomic nervous system in chronic post-cast pain (CPCP) rats. We measured the BP, HR, and baroreflex sensitivity in the resting condition before, during, and after cast immobilization over time. To elucidate additionally the responsiveness to the cold stress, we investigated changes in the BP and HR in response to a low ambient temperature (LT).

## Materials and methods

### Animals

All experiments in this study were approved by the Animal Care Committee of Aichi Medical University and were conducted in accordance with the guidelines for pain research in animals issued by the International Association for the Study of Pain. Male Sprague-Dawley rats (300–350 g) obtained from Japan SLC (Hamamatsu, Japan) were used in this study. Rats after a surgery for telemetric recording were housed individually in plastic cages in a temperature- and relative humidity-maintained room (23.0 ± 0.5°C and 50 ± 10%, respectively) with a 12-h light-dark cycle (lights on at 08:00 h). The animals were allowed access to food and water ad libitum throughout the experiments. During the immobilization period, the animals were free to move in their cages using forelimbs and the non-immobilized hind limb. We attest that every effort has been made to reduce the number of animals used and their suffering as much as possible.

### Cast immobilization

CPCP was induced by a two-week unilateral cast immobilization of one hind limb, according to our previous studies [26, 27]. Briefly, the area from the pelvis to the middle of the hind paw was wrapped using a plaster cast (Plasrun-Gyps E; Alcare, Tokyo, Japan) under sodium pentobarbital anesthesia (50 mg/kg, intraperitoneally). Rats with any sign of circulatory disorders, such as ischemia, congestion, or pressure ulcer due to cast application, or severe cast damage during the cast immobilization period were excluded from the experiments (1 of 10 rats). Five age- and weight-matched untreated rats were assigned as controls.

### Pain behaviors

All behavioral tests were conducted under blinded comparative procedures. Mechanical allodynia of the calf skin and the calf muscles was assessed as previously described [26]. We measured the withdrawal responses to a 20-g von Frey filament stimulation on the medial surface of the calf skin. The stimulus was applied five times (once every 2 s) to the calf skin, and the number of hind limb withdrawal responses was counted. Data are presented as frequency (%) of positive responses. The mechanical pain threshold in the calf muscle was measured with a push-pull gauge algometer (diameter of the probe tip: 2.4 mm; Aikoh Engineering, Osaka, Japan). The calf muscle belly was stimulated by the push-pull gauge with a gradual increase in pressure (10 g/s), and the pressure applied to induce a hind limb withdrawal response was measured. The measurement was performed four times at intervals of 60 s or more, and the median value of the last three measurements was adopted as the withdrawal threshold. During these measurements, each rat was covered from the head to the pelvis with a cloth. These behavioral tests were performed on three different days before cast application, and the average value was used as the baseline.

### Measurements of cardiovascular parameters

#### Telemetric recording of arterial BP and HR

All surgical procedures were performed under anesthesia with sodium pentobarbital (60–70 mg/kg, intraperitoneally) in surgically clean conditions. A telemetry catheter with a BP transducer (Physiotel TA11PA-C40, Data Sciences International, USA) was inserted into the abdominal aorta just below the renal artery and above the bifurcation of the iliac artery. The BP transducer was sutured to the inner surface of the peritoneal wall. Before closing the incision with a suture, a kanamycin sulfate solution (5 mg/ml; Meiji Co., Ltd., Japan) was sprayed into the abdominal cavity. Animals were allowed to recover from the surgery for 2 weeks before starting the data collection. For telemetric recording, a cage with an individual rat was placed on the BP receiver. The sampling procedure was programmed using LabVIEW 7.1 (National Instruments Japan Co., Japan). Consecutive BP pulse waves were processed to measure the systolic arterial blood pressure (SBP) and pulse interval (inversely calculated to instantaneous HR), and the SBP time series (TS_SBP_) was constructed for the frequency analysis as below [30].

#### Frequency analysis of BP fluctuations

In the analysis of BP fluctuation, the “Smooth Spline” function was used in the software R (https://www.r-project.org). The power of each frequency in the TS_SBP_ variability was calculated using a continuous Gabor wavelet transform (frequency resolution, 0.0256 Hz; frequency band, 0.0256–12.8 Hz). The power values within the middle-frequency (MF: 0.33–0.73 Hz) band, which are known to reflect the vascular sympathetic tone [31, 32], were calculated over successive 30-min periods and employed as the MF component.

### Experimental protocols

#### Measurements of cardiovascular parameters during resting conditions

Cardiovascular parameters (SBP, HR, and MF component) during resting conditions were measured in CPCP rats (n = 9) and control rats without cast immobilization (n = 5). Each rat was individually placed in an acrylic cage (205 mm × 300 mm × 130 mm) in a sound-attenuated, temperature- and humidity-controlled room (23.0 ± 0.5°C and 50 ± 10%, respectively). The animals were allowed to acclimatize to this environment for 30 min. The measurements of these cardiovascular parameters were conducted between 10:00 and 16:00 h. In observing the rats’ movements by the investigator, BP data recording was conducted for 30 min, with the rats lying or sitting without strenuous physical activity such as grooming, eating, exploring, or rearing.

These measurements were performed three times across multiple days before the cast application, and the average was used as baseline data before cast application. In CPCP rats, the measurements were performed during the 2-week cast immobilization, and days 1–2, days 3–4, and days 5–6 after cast removal, followed by weekly measurements to 10 weeks. In control rats, the measurements were performed on the day corresponding to weeks 1, 3, 6, and 10 after cast removal.

#### Sympathetic blocker trial

Phentolamine mesylate (PHE; Novartis Pharma, Japan) was used in this study. Each rat was individually placed in an acrylic cage (205 mm × 300 mm × 130 mm) in a sound-attenuated, temperature- and humidity-controlled room (23.0 ± 0.5°C and 50 ± 10%, respectively). After recording baseline data for 20 min, PHE (10 mg/kg) was administered intraperitoneally, which was sufficient to lower the BP and increase the HR within 5 min, lasting at least 60 min after administration. We averaged the 20-min data from 10 to 30 min after administration. The effects of PHE administration on SBP and HR were tested three times, that is, at the time before, during (days 7, 10, or 13), and after cast immobilization (weeks 1, 4, 6, or 8) in CPCP rats.

We determined the baroreflex sensitivity from the SBP and HR responses to PHE administration. Baroreflex sensitivity was calculated as the ratio of the change in HR (ΔHR) divided by the change in SBP (ΔSBP).

#### Effects of a low ambient temperature on SBP and HR

**Fig 1:**
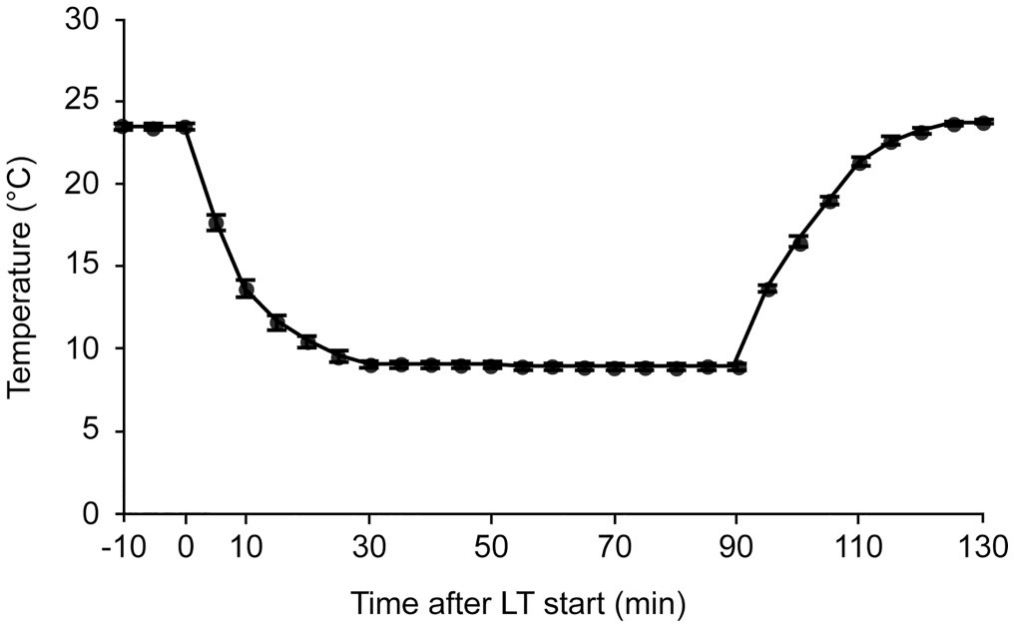
Temperature change of the low ambient temperature (LT) exposure. The average temperature change following LT exposure is shown (mean ± standard error of mean, n = 15). The time after starting LT (min: minutes) is indicated on the horizontal axis.

To expose unstrained rats to an LT for 90 min, each rat was individually kept in an acrylic cage (205 mm × 300 mm × 130 mm) placed inside a Styrofoam box (280 mm × 350 mm × 210 mm) in a sound-attenuated, temperature- and humidity-controlled room (23.0 ± 0.5°C and 50 ± 10%, respectively). After data recording under resting conditions for 60 min, crushed ice was placed around the cage inside the box. Fig 1 shows the average temperature change obtained from 15 trials of LT exposure. The ambient temperature in the cage fell from 23.0 ± 0.5°C to 9.0 ± 0.2°C within 30 min after the crushed ice had been added and then stayed at this level. When the ice was removed, the temperature returned to the baseline level within 25–30 min. The temperature inside the acrylic cage was well-controlled, and the changes were highly reproducible.

With this method, LT-induced changes in cardiovascular parameters were examined before cast application, during cast immobilization on day 4, and weekly in weeks 1 to 10 after cast removal. SBP and HR were averaged over the last 30-min baseline period as baseline values. Changes in SBP and HR were then calculated by subtracting the baseline data from the averaged data during the 90-min LT exposure period. The values are presented as ΔSBP and ΔHR, respectively.

### Statistical analysis

All statistical analyses were performed using JMP^®^ 15 (SAS Institute Inc., USA). Data are presented as the mean ± standard error of mean (SEM). One-way repeated measures ANOVA (one-way ANOVA) followed by post hoc Dunnett’s tests or two-way repeated measures ANOVA (two-way ANOVA) were used to analyze the influence of the cast immobilization on pain behaviors and cardiovascular parameters. To analyze the changes in cardiovascular parameters after PHE administration, the paired *t*-test or one-way ANOVA followed by Dunnett’s tests were employed, as appropriate. A two-sided *p*-value < 0.05 was considered statistically significant.

## Results

### Pain behaviors

**Fig 2:**
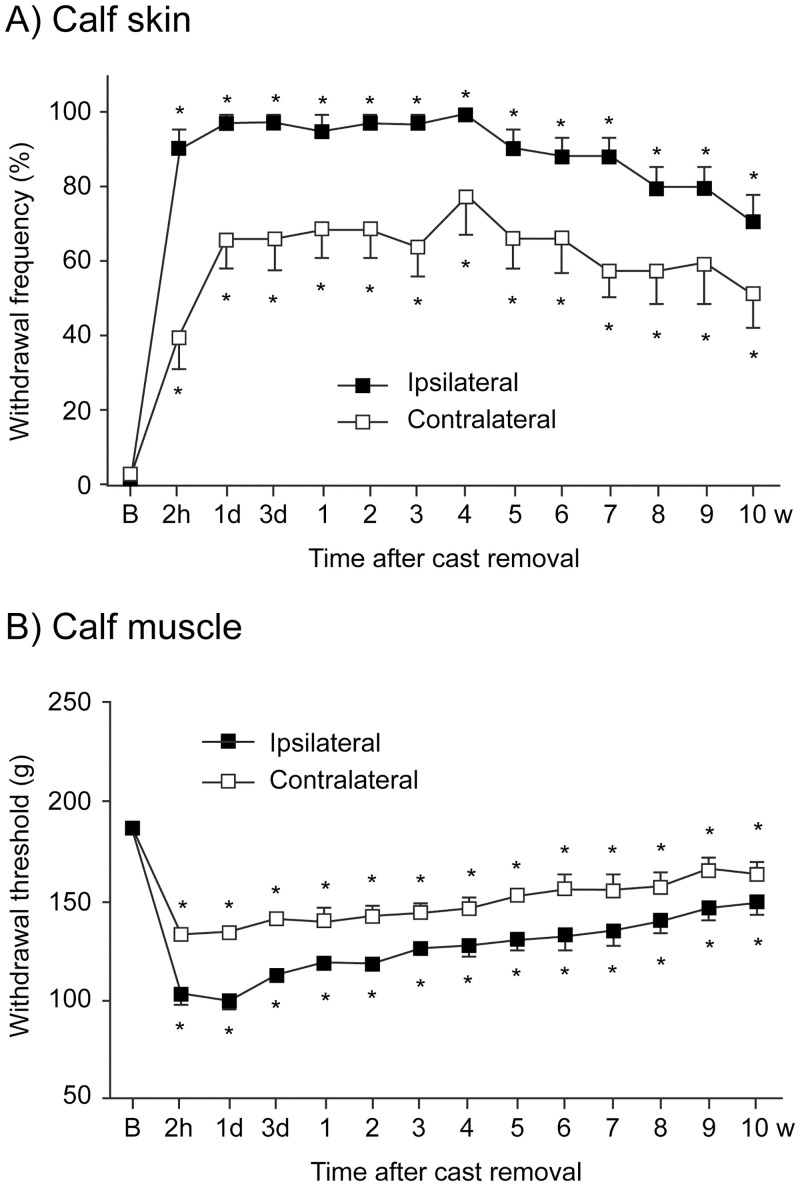
Time course of changes in mechanical allodynia in the calf skin and calf muscles of Chronic Post-Cast Pain (CPCP) rats. The frequency of withdrawal responses to a von Frey filament on the calf skin (A) and the withdrawal threshold to pressure stimuli on the calf muscle (B) in CPCP rats are shown (mean ± SEM, n = 9). The measurement time points are indicated on the horizontal axis (B, before cast application; h, hour; d, day; w, week). *p < 0.05 versus values before cast application (one-way ANOVA and post hoc Dunnett’s tests).

After cast removal, hair loss, redness, and shiny skin of the immobilized area were observed, suggesting local inflammatory changes had occurred [26, 27]. The two-week cast immobilization significantly augmented the frequency of ipsilateral hind limb withdrawal responses to the stimuli by von Frey filaments on the calf skin in CPCP rats (F_(13,104)_ = 37.8, *p* < 0.001, n = 9; Fig 2A). This increased frequency of the response appeared 2 h after cast removal and remained at a significant level for 10 weeks after cast removal. In addition, the augmented frequency of the withdrawal response was observed in the contralateral hind limb of CPCP rats (F_(13,104)_ = 7.5, *p* < 0.001; Fig 2A). These contralateral responses were lower compared to the ipsilateral ones; however, the time-course changes in the withdrawal response were similar on both sides.

Fig 2B shows the changes in the mechanical threshold to pressure stimuli on the calf muscles of CPCP rats. The mechanical withdrawal thresholds decreased bilaterally after cast removal (ipsilateral, F_(13,104)_ = 39.0; contralateral, F_(13,104)_ = 19.1; *p* < 0.001 for both, n = 9). These decreased thresholds reached a minimum within 1 day after cast removal, and the significantly decreased levels lasted for 10 weeks after cast removal, although this effect was gradually reduced.

### Effects of cast immobilization on cardiovascular parameters under resting conditions

**Fig 3:**
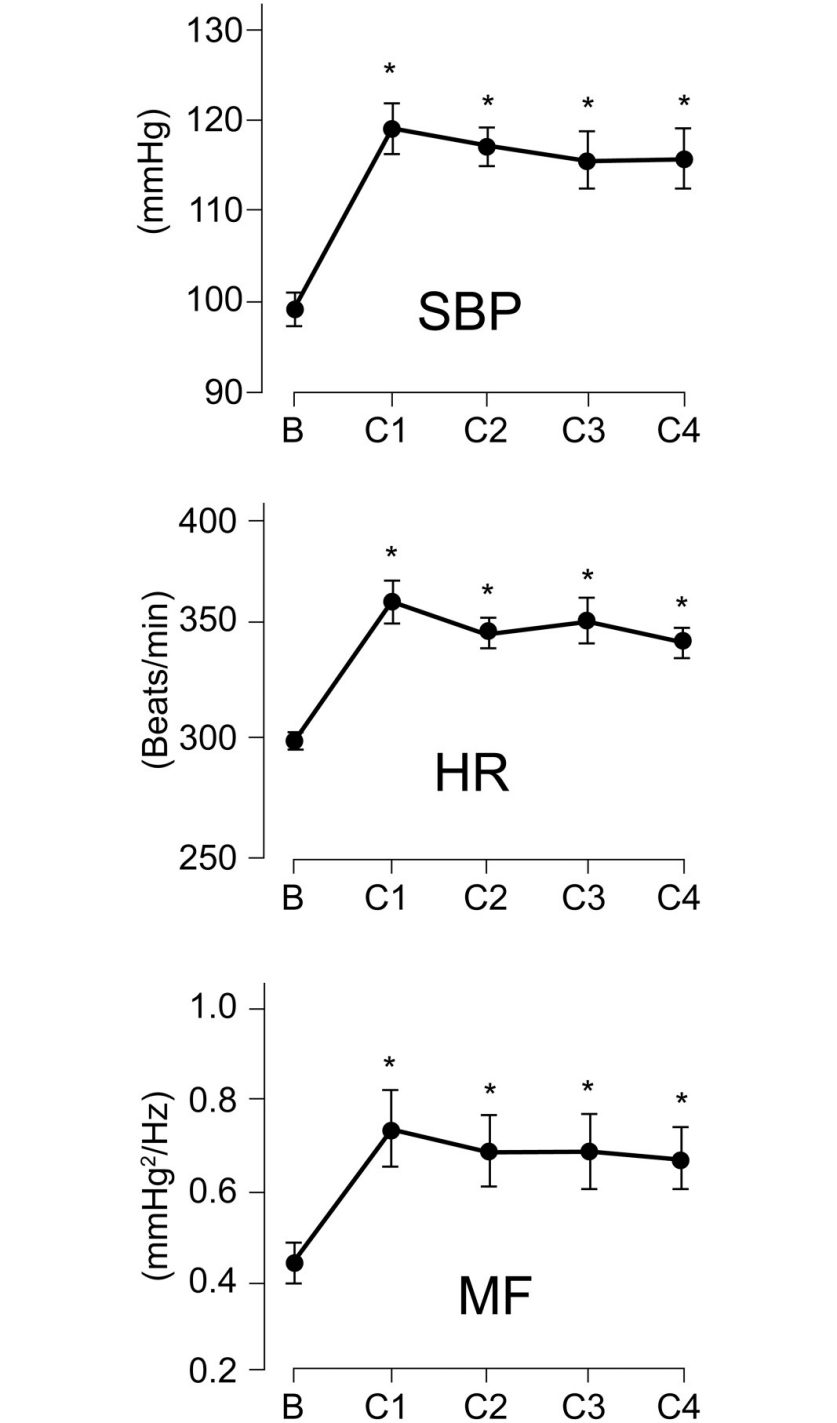
Time courses of changes in cardiovascular parameters during cast immobilization. Systolic arterial blood pressure (SBP; upper), heart rate (HR; middle), and middle-frequency component on the SBP variability spectrum (MF; lower) in chronic post-cast pain (CPCP) rats are shown (mean ± SEM, n = 9). The measurement time points are indicated on the horizontal axis (B, before cast application; C1, days 1–4; C2, days 5–7; C3, days 8–11; C4, days 12–14 after cast application). *p < 0.05 versus values before cast application (one-way ANOVA and post hoc Dunnett’s tests).

**Fig 4:**
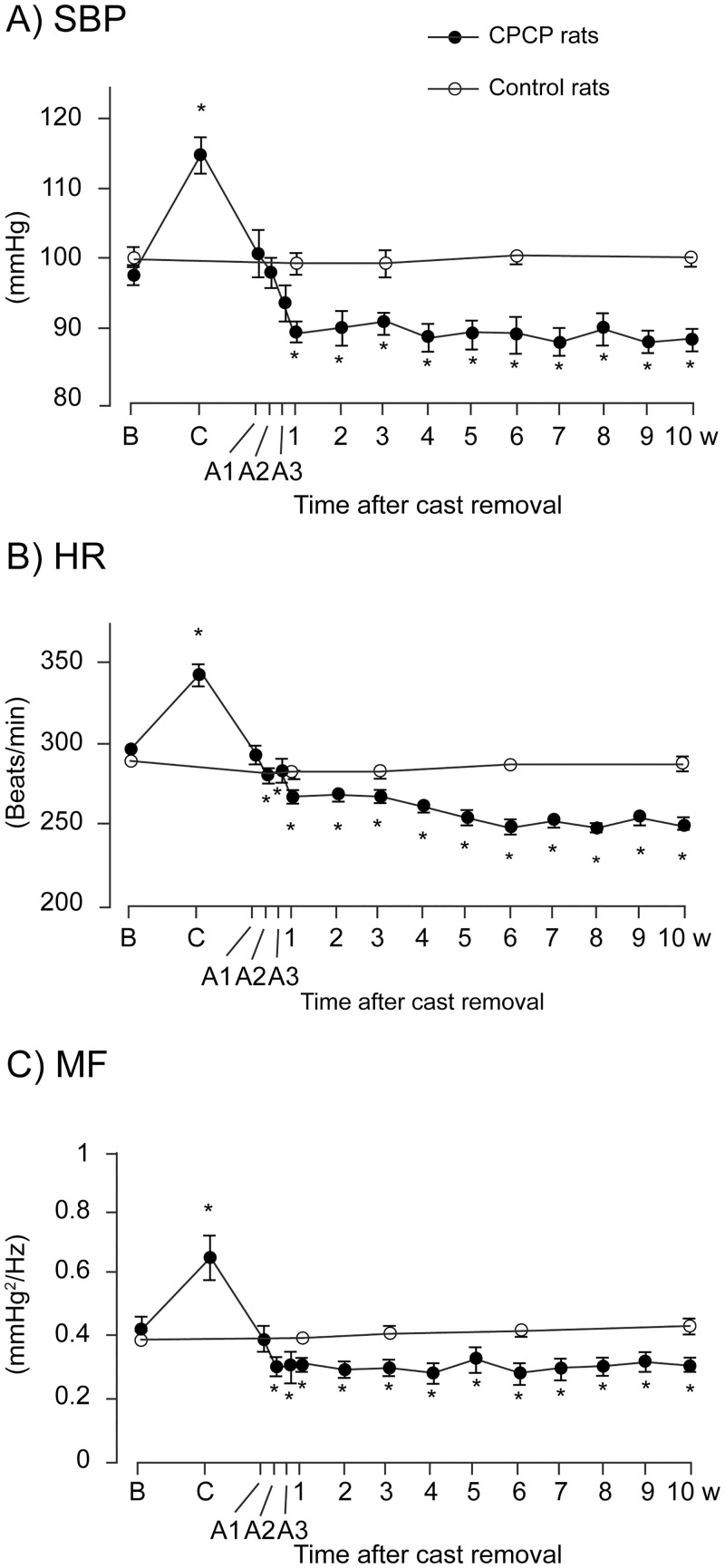
Time courses of cardiovascular parameter changes in resting Chronic Post-Cast Pain (CPCP) rats. Systolic arterial blood pressure (SBP; A), heart rate (HR; B), and MF component on the SBP variability spectrum (MF; C) at rest in CPCP (solid circles; n = 9) and untreated (open circles; n = 5) rats are shown (mean ± SEM). The measurement time points are indicated on the horizontal axis (B, before cast application; C, during cast immobilization; A1, days 1–2; A2, days 3–4; A3, days 5–6 after cast removal; w, weeks). *p < 0.05 versus values before cast application (one-way ANOVA and post hoc Dunnett’s tests). Note that cast immobilization significantly changed these cardiovascular parameters.

The time courses of cardiovascular parameters during cast application in CPCP rats are shown in Fig 3. The SBP, HR, and MF components during the 30-min resting condition were significantly increased as early as time point C1 (days 1–4 after cast application), and these increases remained stable throughout the entire cast period (C2, days 5–7; C3, days 8–11; C4, days 12–14 after cast application). These increases were statistically significant (SBP, F_(4,32)_ = 47.2; HR, F_(4,32)_ = 24.3; MF component, F_(4,32)_ = 18.6; *p* < 0.001 for all, n = 9) throughout the entire cast immobilization period. Fig 4 shows the time courses of cardiovascular parameters in CPCP and control rats. A two-way ANOVA identified a significant main effect of the treatment group in SBP (treatment, F_(1,12)_ = 15.9; time, F_(4,48)_ = 6.7; treatment * time, F_(4,48)_ = 7.4; *p* < 0.01 for all). In control rats, the SBP, HR, and MF components did not change significantly over the entire experimental period (SBP, F_(4,16)_ = 0.3; HR, F_(4,16)_ = 2.2; MF component, F_(4,16)_ = 1.8; *p* > 0.1 for all, n = 5). By contrast, resting CPCP rats exhibited a significant increase in SBP during cast immobilization (Fig 4A), and this increase quickly disappeared after cast removal and stayed at a lower level during the rest of the study period (F_(14,112)_ = 39.1, *p* < 0.001, n = 9). The SBP was significantly higher during cast immobilization (*p* < 0.001), and the SBP values at 1–10 weeks after cast removal were significantly lower than those before cast immobilization (*p* < 0.001 for all). Additionally, in HR, a two-way ANOVA revealed a significant main effect of the treatment group (treatment, F_(1,12)_ = 18.3; time, F_(4,48)_ = 32.2; treatment * time, F_(4,48)_ = 28.7; *p* < 0.01 for all). The HR in resting CPCP rats was also significantly increased during cast immobilization and decreased after cast removal (F_(14,112)_ = 66.6, *p* < 0.001; Fig 4B), the time course of which was similar to that of SBP. The HR was significantly higher during cast immobilization (*p* < 0.001) and significantly lower at 1–10 weeks after cast removal than before cast application (*p* < 0.001 for all). Significant treatment effects were identified in the MF component as well (treatment, F_(1,12)_ = 6.6; time, F_(4,48)_ = 3.8; treatment * time, F_(4,48)_ = 6.6; *p* < 0.05 for all). The MF component was also significantly changed by the cast immobilization (F_(14,112)_ = 15.7, *p* < 0.001; Fig 4C). The time course of the MF component was similar to those of the SBP and HR. Taken together, these results suggest that cardiovascular sympathetic activity increased transiently during cast immobilization, decreased within a few days after cast removal, and stayed at a lower level thereafter.

### Effects of PHE on cardiovascular parameters

**Fig 5:**
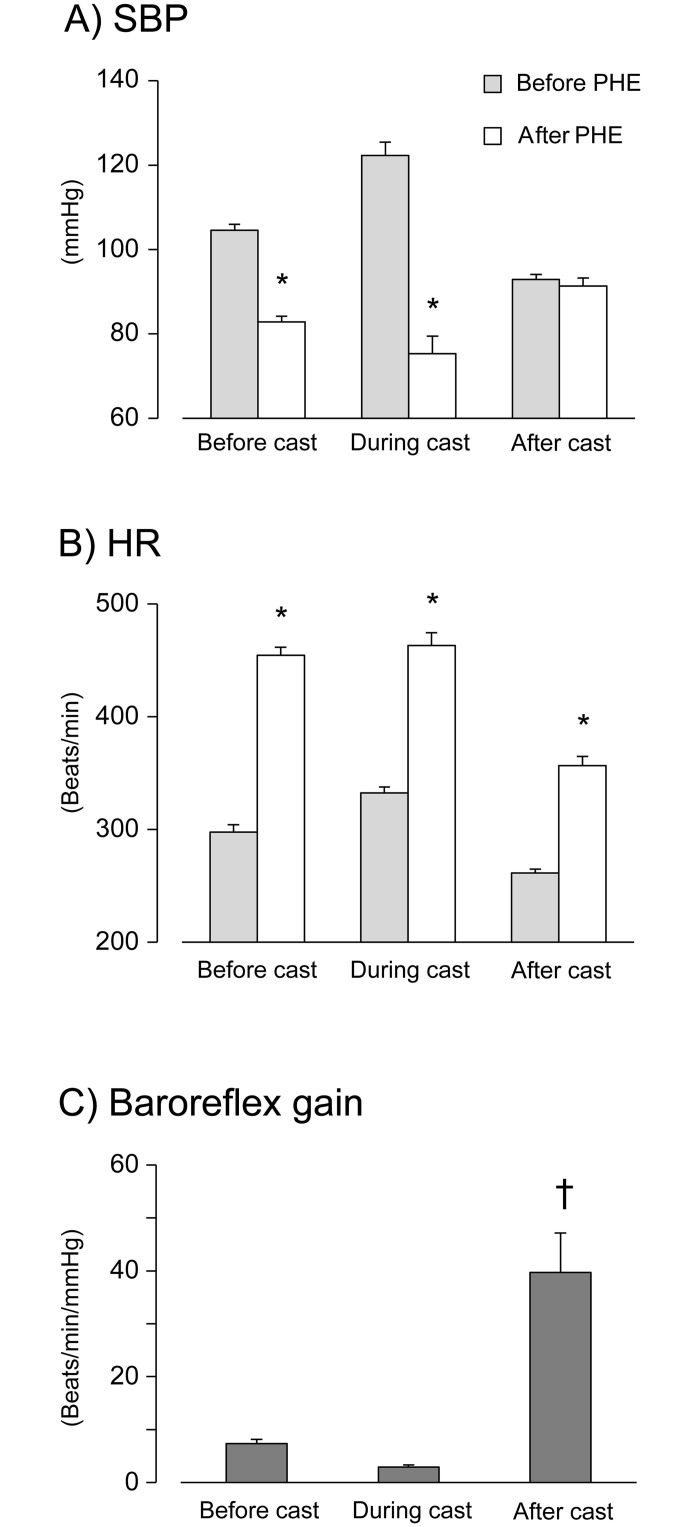
Responses of cardiovascular parameters to phentolamine (PHE) administration before, during, and after cast immobilization. Changes in the systolic arterial blood pressure (SBP; A) and the heart rate (HR; B) by PHE administration before, during and after cast application are shown (n = 5, mean ± SEM). (C) indicates the baroreflex gain determined by phentolamine as an indicator of baroreflex sensitivity. Note that there were significantly higher baroreflex gains after cast application compared to that before and during cast application. *p< 0.05, compared to before PHE with after PHE values (paired *t*-test). †*p*< 0.05, compared to that before cast application (one-way ANOVA and Dunnett’s post hoc tests).

The raw data of the degree of changes in SBP and HR to PHE administration in each CPCP rat are shown in S1 Fig. This figure shows that the degree of changes in SBP by PHE administration clearly decreased 1–8 weeks after cast removal. Therefore, we averaged the data for each period (before cast, during cast, and after cast), as shown in Fig 5. In Fig 5A, PHE significantly decreased SBP in both the periods, before and during cast immobilization (before, *p* < 0.001; during, *p* = 0.001), suggesting that the vascular sympathetic control was not influenced by cast immobilization. By contrast, PHE did not lower the SBP after cast removal (*p* = 0.21), suggesting that vascular sympathetic control in CPCP rats was significantly suppressed after cast removal.

The comparison of the PHE effects on the HR before, during, and after cast immobilization of CPCP rats is shown in Fig 5B. PHE significantly increased HR before, during, and after the cast immobilization period (*p* < 0.001 for all). This suggests that the cast immobilization did not influence cardiac sympathetic control in CPCP rats. These difference in PHE sensitivity between SBP (i.e., a vascular parameter) and HR (i.e., a cardiac parameter) after the cast immobilization period are confirmed by Fig 5C, in which the baroreflex gain, taken as the HR increase divided by the SBP reduction after PHE administration, is significantly increased after cast removal compared to the time before cast application (F_(2,8)_ = 20.2, *p* < 0.001).

### Changes in cardiovascular parameters during LT exposure

**Fig 6:**
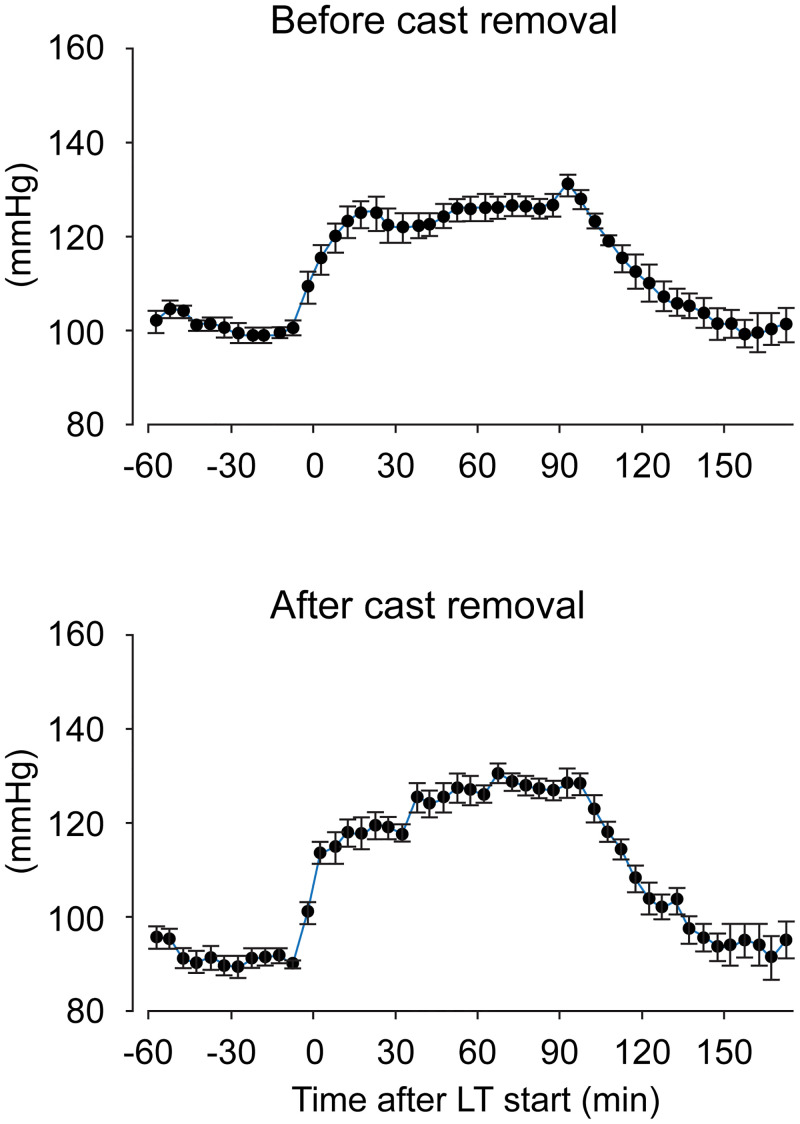
Mean systolic arterial blood pressure (SBP) responses to low ambient temperature (LT) exposure before cast application and 6 weeks after cast removal. The time after starting LT (min: minutes) is shown on the horizontal axis. Data are presented as the mean ± SEM (n = 9).

**Fig 7:**
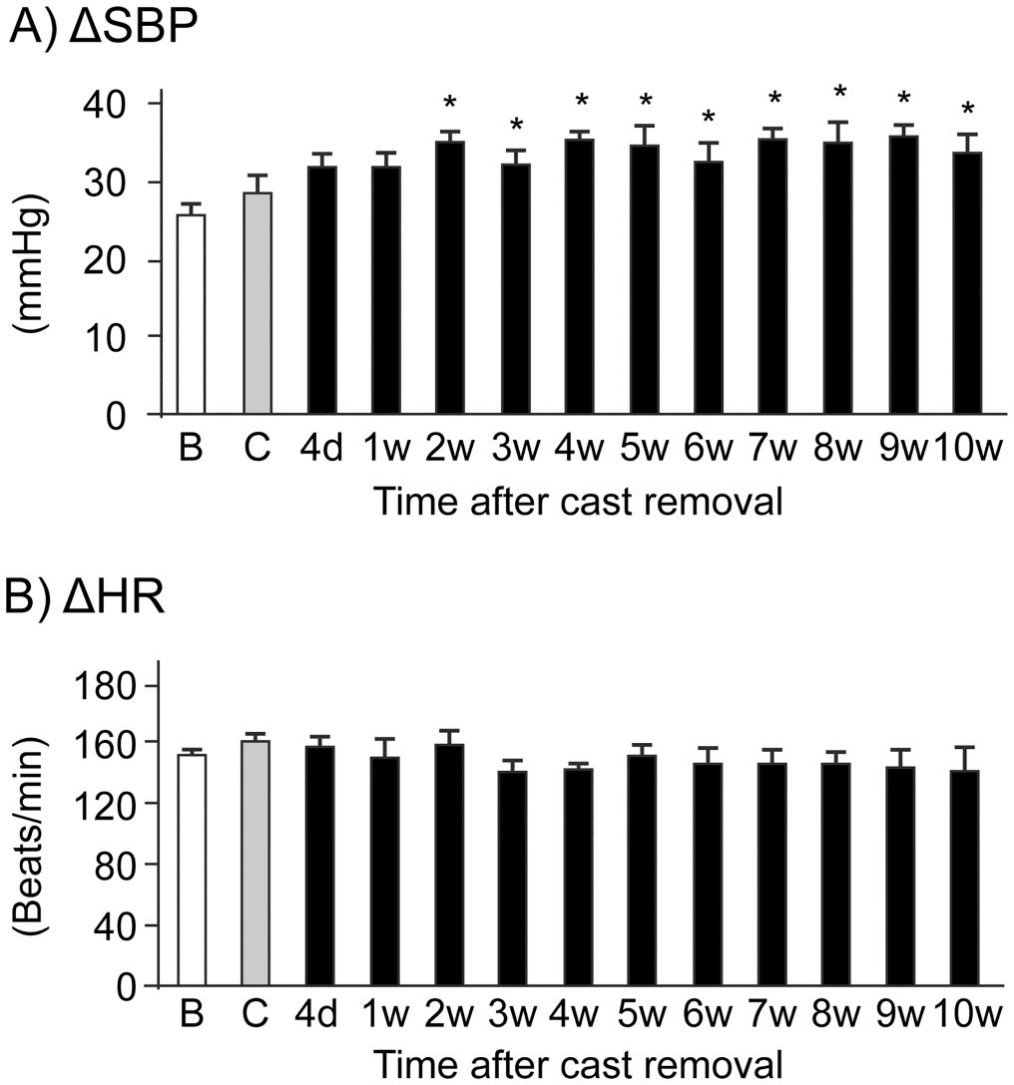
Time courses of systolic arterial blood pressure (SBP) and heart rate (HR) changes to low ambient temperature (LT) exposure. Responses of cardiovascular parameters to LT exposure are shown as the changes in SBP (A) and HR (B). The values were calculated by subtracting the data before LT exposure from the averaged data during LT exposure. The measurement time points are indicated on the horizontal axis (B, before cast application; C, during cast immobilization; d, day; w, week). Data are presented as the mean ± SEM (n = 6). *p < 0.05 versus values before cast application (one-way ANOVA and post hoc Dunnett’s tests).

We examined the effects of LT exposure (i.e., a sympathetic stimulant) on the cardiovascular parameters in CPCP rats Fig 6 shows the SBP changes during LT exposure before cast immobilization and 6 weeks after cast removal (n = 9). The SBP increase in rats 6 weeks after cast removal appears larger compared to that in rats before cast application, suggesting that SBP sensitivity to LT exposure was increased after cast removal. This is confirmed by Fig 7A, showing the time course of ΔSBP changes after cast removal (n = 6). The parameter ΔSBP did not change during cast immobilization but was significantly increased after the cast had been removed; this increase was maintained for up to 10 weeks after cast removal (F_(12,60)_ = 3.2, *p* = 0.001). By contrast, LT exposure did not induce any significant changes in ΔHR (F_(12,60)_ = 0.9, *p* = 0.55).

All dataset in our study are shown in S1 File.

## Discussion

This study’s behavioral observations confirm our original findings [26, 27] that unilateral cast immobilization of a hind limb for 2 weeks in rats produces long-lasting pain behaviors, including mechanical allodynia, in the bilateral hind limbs. This study further demonstrated that the two-week cast immobilization also induced changes in cardiovascular parameters in the resting condition and increased the sensitivity to an α-adrenoceptor antagonist, PHE, and a sympathetic stimulant, LT exposure.

This study demonstrated that the cardiovascular parameters SBP, HR, and MF component were significantly increased under resting conditions during cast immobilization and remained at elevated levels during the two-week cast immobilization period in CPCP rats (Figs 3 and 4). The increase in SBP, an indicator of peripheral sympathetic activity, was considerably suppressed by intraperitoneal PHE administration (Fig 5), suggesting the SNS activation via α-adrenoceptors during cast immobilization. Some stress induced by restraint or additional weight-bearing may be involved in this increase, but the factor responsible for this SNS activation could not be determined in the present study. The result of a decrease in elevated SBP by PHE administration during cast immobilization implies that it is quite unlikely to apply compression on the vessel mechanically by the cast. Moreover, the increase in SBP during cast immobilization gradually decreased over 1 week after cast removal (Fig 4), suggesting that the increases in cardiovascular parameters during cast immobilization are not due to cast-induced mechanical compression of the vessels.

Our study also demonstrated that after cast removal, the cardiovascular parameters under resting conditions were significantly decreased below baseline in CPCP rats. After cast removal, the cardiovascular parameters SBP, HR, and MF component gradually decreased and reached a level lower than baseline within 1 week, and these significantly lower levels lasted for over 10 weeks after cast removal (Fig 4). Furthermore, PHE administration did not induce a decrease in SBP after cast removal, unlike before and during cast immobilization (Fig 5). These results suggest that there is less involvement of α-adrenoceptors in the maintenance of BP after cast removal, as well as a decrease in SNS function, that is, an SNS dysfunction under resting conditions in CPCP rats.

Ohmichi et al. [26] showed that a sciatic nerve block in the immobilized hind limb at 3–8 weeks after cast removal did not suppress the mechanical allodynia in the contralateral side. These authors hypothesized the involvement of plastic changes in central pain pathways. It has been shown that, in some patients with chronic pain, local sympathetic nerve block provides substantial or complete pain relief, suggesting that sympathetic activity may be involved in these pain conditions [10]. However, it is commonly understood that sympathetic nerve activity in the affected extremity of CRPS patients is not increased [10–12]. In addition, plasma noradrenaline levels in the affected extremities of CRPS patients are significantly lower than in the unaffected limbs [13–15]. Our study findings suggest that the reduced sympathetic activity in rats exposed to chronic pain conditions seems to be similar to that in patients with chronic pain.

Some studies have shown that in chronic pain conditions, SNS disturbances were observed in both the affected and the unaffected limb [19–21], suggesting that there is no regional but a systemic sympathetic dysfunction in chronic pain conditions. Recently, some studies in animal models of chronic pain have investigated cardiovascular function using telemetry monitoring [7, 23–25]. Jin et al. [7] demonstrated for the first time in conscious animals that rats with unilateral CCI of the sciatic nerve as a chronic pain model exhibited significant increases in arterial BP and HR at the beginning of the pathological condition (up to 7 days following CCI surgery) and then returned to the levels before surgery or below. Our results resemble the shift in these results, although the animal models of chronic pain differed.

Homeostasis in the circulatory system depends on neural regulation driven by the autonomic nervous system. Among the assessments of autonomic nervous system functions, baroreflex sensitivity as an index of circulatory system modulation can be used. The baroreflex sensitivity values after cast removal were significantly higher than those before and during cast immobilization (Fig 5C). This was observed because PHE induced no significant decrease in SBP but increased the HR after cast removal (Fig 5A and 5B). Our data show that baroreflex sensitivity is enhanced following cast immobilization, and this increased baroreflex sensitivity may imply a dysfunction of the cardiovascular regulation involving the central nervous system. Gemes et al. [23] demonstrated that the baroreflex sensitivity in an animal model of chronic pain after spinal nerve ligation was diminished compared to that in the control group. Since their study differs from ours regarding animal models and assessment methods, their results cannot be directly compared with our findings.

To shed light on stress responsiveness, we also investigated the cardiovascular responses to LT exposure. In our previous study, we confirmed that the increases in SBP and MF components by LT exposure were suppressed by PHE administration [30]; therefore, LT exposure in the present study is considered appropriate to examine sympathetic activation in conscious rats. Our current results showed that the responsiveness of SBP to LT exposure was increased after cast removal when the SNS activity might be decreased under resting conditions (Figs 6 and 7). One reason for this result may be the hypersensitivity of adrenoceptors to SNS neurotransmitters. Kurvers et al. [33] demonstrated that the basal skin blood flow in the CCI model is increased until day 5 and decreased during days 7–28. Blocking the impulse propagation in the injured sciatic nerve at day 28 did not change the reduced skin blood flow. The authors of this study hypothesized that constriction injury of the sciatic nerve reduces the sympathetic vasoconstrictor activity, which may subsequently lead to hypersensitivity of skin microvessels to noradrenaline. Based on a study of vasoconstrictor responses to intravenous noradrenaline administration, Arnold et al. [6] suggested the hyperresponsiveness of vascular adrenoceptors to noradrenaline in patients with reflex sympathetic dystrophy. Recently, some studies have indicated that augmented responsiveness to noradrenaline may be dominant in chronic pain conditions rather than an increased α-adrenoceptor density in peripheral blood vessels [34–36]. The enhanced responsiveness of α-adrenoceptors to peripheral nociceptive fibers has also been demonstrated in an animal model of neuropathic pain [37]. Assessment of immunohistochemical staining of peripheral tissues is warranted in order to better understand our results regarding the responsiveness to LT exposure in CPCP rats.

Our study was limited by its control group. Previous studies [38, 39] have shown that the cardiovascular parameters used did not change throughout several weeks in normal rats (the same-strain and same-age rats were used). We measured our control rats every 2–3 weeks. With regard to a sympathetic blocker trial, we did not set a control group. In our preliminary experiment, we investigated the effects of PHE administration on the SBP at various ages in normal rats (S1 Table). These data indicated that the response of SBP to PHE was similar, regardless of the age of the normal rats. We also compared the degree of SBP response between the first and second administration of PHE in normal rats (S2 Table). There were no obvious differences in the SBP response between the two administrations. Hence, we did not use a control group for the PHE experiments. However, the repeated measurements of the stressful events such as LT exposure may have affected this study’s outcomes. We also did not evaluate the direct relationship between pain and sympathetic nervous activity. Further studies with direct recordings of nociceptive neuron discharges in response to sympathetic stimulation may help to clarify the involvement of nociceptive-sympathetic crosstalk in our CPCP model.

In conclusion, we found that, in the CPCP model, cardiovascular parameters were increased during cast immobilization and decreased below baseline until 10 weeks after cast removal. These results suggest that sympathetic activity was augmented by immobilization and were subsequently lower than the normal level when persisting pain behaviors continued. After cast removal, our data also pointed to baroreflex sensitivity dysfunction and hyperresponsiveness of SBP to LT exposure. Overall, this in the CPCP rat model implies systemic sympathetic dysfunction in chronic pain conditions under both resting and activated conditions by external stimuli.

## Supporting information

S1 Fig Changes in the cardiovascular parameters to phentolamine (PHE) in Chronic Post-Cast Pain (CPCP) rats. (TIF)

S1 File Dataset. (PDF)

S1 Table Responses of systolic arterial blood pressure (SBP) to phentolamine (PHE) at various ages in normal rats. (PDF)

S2 Table Responses of systolic arterial blood pressure (SBP) to double administration of phentolamine (PHE) in normal rats. (PDF)

S1 Checklist (PDF)
