# Supplementary figures and images for: Changes in cardiovascular parameters in rats exposed to chronic widespread mechanical allodynia induced by hind limb cast immobilization
Source: PLoS One. 2021 Jan 19;16(1):e0245544. doi: 10.1371/journal.pone.0245544 (PMC7815128; doi:10.1371/journal.pone.0245544)

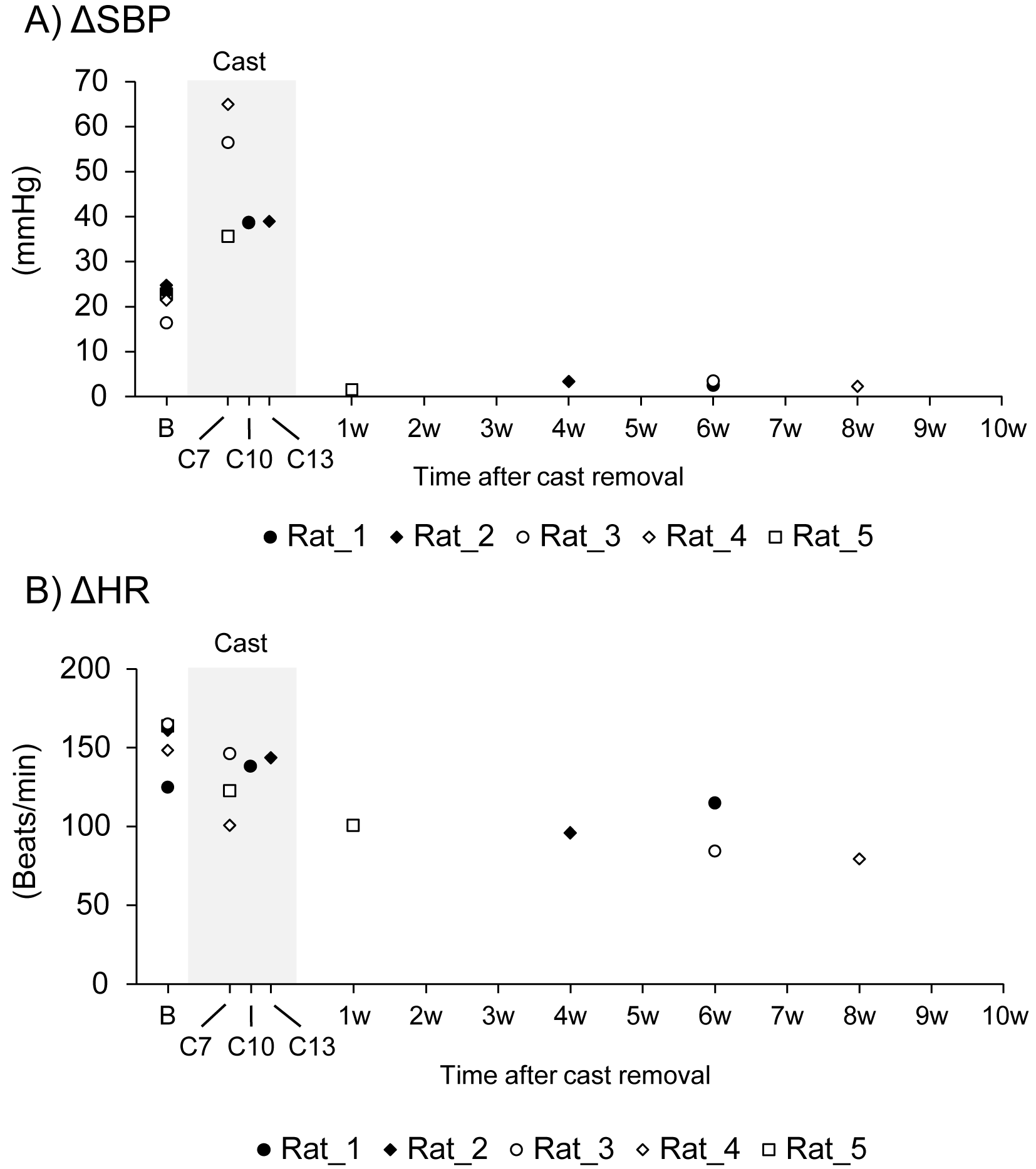

Supplement: S1 Fig — (TIF) [file pone.0245544.s001.tif]
