# Supplementary material for: Changes in cardiovascular parameters in rats exposed to chronic widespread mechanical allodynia induced by hind limb cast immobilization
Source: PLoS One. 2021 Jan 19;16(1):e0245544. doi: 10.1371/journal.pone.0245544 (PMC7815128; doi:10.1371/journal.pone.0245544)
Supplement: S1 File — (PDF) [file pone.0245544.s002.pdf]

Data (Fig 1)

Time	1	2	3	4	5	6	7	8	9	10	11	12	13	14	15
−10	24.9	24.7	23.4	23.0	23.2	22.8	23.3	22.7	24.6	22.5	22.9	22.8	22.9	23.7	24.6
	24.6	24.6	23.4	23.0	23.3	22.8	23.2	22.8	24.7	22.4	22.9	22.5	22.9	23.7	24.6
0	24.7	24.6	23.5	23.0	23.4	22.7	23.3	22.8	24.6	22.5	23.0	22.9	22.9	23.7	24.4
	20.4	18.7	17.9	19.7	17.5	16.2	15.4	15.6	19.3	18.5	15.6	21.1	17.8	15.6	16.5
10	16.5	15.4	14.7	15.6	11.6	12.5	12.5	11.6	15.6	12.8	12.9	16.2	12.8	11.9	12.9
	15.9	13.6	13.0	12.5	11.0	10.6	10.5	10.2	11.0	10.5	10.5	13.6	10.5	10.5	11.0
20	13.9	13.0	10.9	11.9	9.9	9.3	9.3	9.5	10.1	9.9	9.3	11.4	9.9	9.3	9.5
	12.5	11.8	10.5	10.1	8.8	8.5	8.5	8.5	9.3	9.1	9.1	9.9	9.1	8.5	9.3
30	11.0	10.3	10.3	9.5	8.8	8.2	8.3	8.3	9.0	8.8	8.7	9.5	9.0	8.3	8.9
	10.9	10.2	10.2	9.4	8.8	8.1	8.5	8.2	9.0	8.7	8.7	9.5	8.9	8.5	9.0
40	11.0	10.2	10.2	9.4	8.9	8.1	8.5	8.2	9.0	8.8	8.6	9.4	8.8	8.5	8.9
	10.6	10.3	10.2	9.4	9.0	8.3	8.6	8.3	9.1	8.8	8.6	9.5	8.8	8.0	8.6
50	10.4	10.4	10.3	9.3	8.9	8.0	8.5	8.2	9.3	8.8	8.6	9.1	9.4	7.8	8.6
	10.2	10.3	10.2	9.3	8.5	7.9	8.5	8.2	9.3	8.8	8.7	8.8	9.0	8.3	8.6
60	10.1	10.1	10.2	9.5	8.4	8.1	8.6	8.3	9.1	8.6	8.7	8.8	8.8	8.4	8.7
	10.1	9.9	10.2	9.3	8.4	8.1	8.5	8.3	9.1	8.6	8.7	9.0	8.8	8.4	8.7
70	10.1	9.8	10.3	9.3	8.1	8.1	8.5	8.3	9.3	8.6	8.7	9.3	8.5	8.2	8.5
	9.9	9.8	10.3	9.3	7.9	8.6	8.4	8.5	9.6	8.6	8.7	9.9	8.3	8.0	8.5
80	9.9	9.8	10.3	9.4	7.9	8.2	8.5	8.5	9.5	8.6	8.6	9.4	8.3	8.1	8.7
	9.9	9.7	10.5	9.4	8.1	8.0	8.5	8.5	9.5	8.6	8.8	9.5	8.4	8.1	8.7
90	9.8	9.8	10.5	9.5	8.1	7.9	8.5	8.5	9.6	8.4	8.8	9.4	8.5	8.2	8.7
	12.8	12.9	15.9	15.4	12.9	12.9	13.6	12.8	13.9	12.8	12.8	15.9	13.8	13.8	13.0
100	15.4	15.6	18.5	17.9	16.2	15.4	16.2	15.6	18.5	15.5	15.5	18.5	17.8	15.6	15.5
	17.9	19.3	19.7	21.1	19.3	17.5	18.5	18.7	21.1	18.7	17.5	19.3	19.1	18.5	19.3
110	20.4	21.3	21.3	22.9	21.1	20.4	21.1	21.5	22.8	21.3	20.4	22.8	20.4	21.5	21.1
	21.3	23.4	22.8	23.7	22.5	22.0	22.5	22.5	23.5	22.8	22.5	23.5	21.1	22.9	22.5
120	21.5	24.2	23.0	24.4	22.8	22.7	23.0	23.7	24.2	23.2	22.9	24.2	22.0	23.7	22.9
	23.2	24.0	23.3	24.2	23.4	23.4	23.2	24.0	24.4	23.3	24.1	24.4	22.5	24.1	23.7
130	23.4	24.0	23.3	24.2	23.7	23.7	23.4	24.1	24.2	23.7	24.4	24.2	22.7	24.2	23.7

Data (Fig 2)

Calf skin	B	2h	1d	3d	1w	2w	3w	4w	5w	6w	7w	8w	9w	10w
Contra	0.0	5	5	5	4	4	3	5	4	5	3	3	5	2
Contra	0.7	2	2	2	3	3	3	2	2	2	2	1	1	1
Contra	0.5	1	2	5	2	5	5	5	2	4	3	2	2	3
Contra	0.0	1	3	3	2	2	4	5	5	5	4	5	5	4
Contra	0.0	1	4	5	4	4	4	5	5	2	3	3	3	2
Contra	0.3	2	5	3	5	5	4	5	4	5	5	5	5	5
Contra	0.0	3	4	3	3	3	3	4	3	3	2	2	3	2
Contra	0.0	2	2	2	3	2	2	3	3	2	2	2	2	2
Contra	0.0	1	3	2	5	3	1	1	2	2	2	3	1	2
Ipsi	0.0	5	5	5	5	4	5	5	5	5	5	3	5	3
Ipsi	0.3	4	5	5	5	5	4	5	3	3	3	3	3	3
Ipsi	0.0	5	5	5	5	5	5	5	4	5	4	3	3	3
Ipsi	0.0	5	4	5	3	5	5	5	5	5	5	4	4	5
Ipsi	0.3	5	5	5	5	5	5	5	5	5	5	4	3	2
Ipsi	0.0	4	5	5	5	5	5	5	5	4	4	5	4	5
Ipsi	0.0	5	5	5	5	5	5	5	5	4	5	5	5	5
Ipsi	0.0	5	5	5	5	5	5	5	5	5	4	4	4	3
Ipsi	0.0	3	5	4	5	5	5	5	4	4	5	5	5	3

Calf muscle	B	2h	1d	3d	1w	2w	3w	4w	5w	6w	7w	8w	9w	10w
Contra	177.3	154.5	151.2	145.3	156.6	156	158.9	151.2	161.3	171.2	134.7	167	176.4	172.2
Contra	189.2	139.8	133.1	151.6	149.8	151.6	153.3	168.7	166	176.2	187.6	185.3	191.5	184
Contra	190.7	147.2	135.1	147.3	165.1	170.3	162.2	167.2	156.3	178.8	193.1	190.1	187.7	197.4
Contra	184.3	125.4	129.7	136.6	128.8	128	123.1	122.5	129.7	129	126.5	136.4	132.4	137.1
Contra	179.5	129.6	131.1	138.9	141.3	137.8	145	127.7	140.2	131.9	131.4	152.1	151.7	158.3
Contra	189.0	127.6	135.5	129.4	129.6	143	142.7	145.5	161.2	167.7	170	150.2	172	156
Contra	181.8	113	128.5	137.5	107.4	122.2	124.2	124.2	140	127.8	139	128.4	140.1	141
Contra	189.0	130.2	131.7	138.1	145.2	139.3	139	148	151.9	161.3	152.4	155.5	160.4	163.9
Contra	185.7	115.4	123	133.3	127	124	137.7	151	156.4	152.2	153	141.1	166.2	147.2
Ipsi	176.7	118.3	108.2	112.2	134.6	128.9	137.9	129.9	146.1	127.8	98.4	138.8	144.7	153.3
Ipsi	188.0	119.1	110	126.2	126.1	128.9	137.8	135.1	140	160.1	160.2	171.4	173.1	168
Ipsi	185.0	111.6	104.2	116.1	130.1	138.2	140.2	148.5	133.4	152.2	167	166.4	164.9	170.1
Ipsi	185.9	88	94	106	109.1	101.3	97.8	99.9	107	99.3	110.7	115.6	112.4	109.3
Ipsi	185.7	108.6	88.7	115	126.4	116	130.1	119.1	111.2	106.3	130.1	131	150.3	139.7
Ipsi	183.5	95.1	97.9	101.1	106.2	122	124.4	125.2	144.2	141	139	150	159.3	154.1
Ipsi	185.1	70.8	84.1	109	97.2	102.4	110.2	111.9	119	121	124.2	109.9	118	136.1
Ipsi	193.2	102.5	88.7	97.3	116.2	103.2	122	125.4	120.1	130	141.1	141.2	140	155.5
Ipsi	183.3	103.2	113.8	124.1	112.2	123.1	129.4	140	143.3	143.8	133	130.5	148	152.1

Data (Fig 3)

	B	C1	C2	C3	C4
SBP	92.7	108.6	110.6	111.5	105.2
SBP	101.3	120.9	120.7	124.1	125.6
SBP	106.4	132.8	122.1	125.7	124.7
SBP	103.4	121.1	120.8	114.6	113.2
SBP	90.8	111.1	109.1	96.5	99.9
SBP	97.6	111.1	110.5	110.3	110.6
SBP	95.7	111.6	112.4	111.1	113.3
SBP	102.0	123.6	118.5	118.0	118.3
SBP	98.0	125.3	122.7	122.5	126.3

	B	C1	C2	C3	C4
HR	296.4	380.2	370.5	404.9	351.8
HR	310.3	379.4	355.1	395.4	377.2
HR	289.7	335.9	324.1	329.0	336.1
HR	308.9	408.4	368.2	344.4	341.4
HR	299.3	354.9	348.3	335.5	335.8
HR	299.9	337.8	335.1	333.2	342.0
HR	297.6	342.4	349.7	340.6	339.1
HR	288.1	337.4	326.0	331.7	308.8
HR	288.8	332.2	315.5	330.6	325.7

	B	C1	C2	C3	C4
MF	0.288	0.603	0.607	0.577	0.667
MF	0.313	0.472	0.450	0.495	0.539
MF	0.327	0.555	0.416	0.441	0.471
MF	0.555	0.817	0.855	0.710	0.659
MF	0.399	0.646	0.546	0.458	0.487
MF	0.524	0.820	0.732	0.761	0.645
MF	0.287	0.474	0.501	0.511	0.522
MF	0.556	1.240	1.048	1.065	1.067
MF	0.610	0.888	0.952	1.037	0.876

Data (Fig 4)

CPCP									
	SBP	SBP	SBP	SBP	SBP	SBP	SBP	SBP	SBP
B	92.7	101.3	106.4	103.4	90.8	97.6	95.7	102.0	98.0
C	108.6	122.2	125.9	117.5	106.0	108.9	110.3	118.2	121.1
A1	87.7	103.9	113.7	106.5	86.8	93.0	101.0	103.6	111.8
A2	92.2	101.9	102.6	100.5	84.6	94.8	95.5	97.1	107.9
A3	84.8	102.0	97.1	97.2	83.3	90.3	93.6	91.7	105.5
1w	88.8	94.6	94.7	91.0	83.3	86.4	85.8	90.0	94.1
2w	82.8	98.3	97.4	100.1	81.4	81.7	88.5	92.1	89.9
3w	83.2	95.6	96.5	95.3	90.4	88.7	89.9	90.3	89.0
4w	81.0	86.6	97.7	95.5	82.7	91.9	84.6	90.9	88.3
5w	84.3	95.6	94.8	95.7	79.1	91.3	85.6	90.1	87.1
6w	79.4	94.8	98.0	99.2	85.2	91.9	78.4	92.1	85.0
7w	81.9	93.1	95.4	94.5	79.3	85.0	87.3	91.7	86.3
8w	83.0	95.8	100.4	96.3	80.7	92.7	87.8	89.7	83.9
9w	82.8	93.3	96.0	91.9	85.8	85.3	83.5	88.9	87.2
10w	80.9	89.1	94.8	94.0	80.1	90.6	88.9	92.6	86.7
	HR	HR	HR	HR	HR	HR	HR	HR	HR
B	296.4	310.3	289.7	308.9	299.3	299.9	297.6	288.1	288.8
C	372.9	371.5	328.0	353.9	345.8	337.5	339.6	327.0	322.4
A1	281.2	323.8	315.1	290.8	282.3	283.5	303.5	274.1	288.4
A2	287.8	301.9	282.7	289.3	275.1	264.8	284.0	261.9	287.0
A3	270.9	311.1	287.7	300.4	275.0	278.0	284.8	242.3	305.7
1w	266.7	285.3	259.2	281.1	272.6	263.1	267.2	251.3	268.1
2w	257.5	284.1	269.1	278.3	269.5	265.1	272.8	265.2	254.3
3w	269.6	280.6	268.6	272.3	281.4	270.1	250.3	261.1	260.7
4w	256.1	280.1	260.9	271.0	269.1	263.5	252.2	260.2	245.3
5w	252.2	275.0	261.7	266.2	243.7	243.0	257.4	254.8	242.1
6w	237.9	272.2	251.6	272.4	254.3	256.7	230.2	233.4	240.7
7w	252.9	270.7	247.8	265.6	247.6	257.5	238.7	246.1	250.0
8w	243.9	262.1	254.3	267.0	245.2	244.8	241.7	242.4	237.6
9w	251.9	257.5	244.4	267.0	257.3	248.5	272.5	249.7	243.1
10w	240.2	261.0	253.0	268.1	255.3	246.2	249.7	252.9	235.0
	MF	MF	MF	MF	MF	MF	MF	MF	MF
B	0.288	0.313	0.327	0.555	0.399	0.524	0.287	0.556	0.610
C	0.619	0.442	0.462	0.723	0.564	0.711	0.474	1.063	0.908
A1	0.283	0.367	0.338	0.469	0.281	0.370	0.351	0.449	0.694
A2	0.416	0.217	0.251	0.326	0.369	0.199	0.236	0.349	0.443
A3	0.158	0.282	0.166	0.309	0.210	0.373	0.330	0.301	0.610
1w	0.286	0.292	0.265	0.344	0.246	0.368	0.214	0.340	0.432
2w	0.235	0.208	0.199	0.446	0.252	0.346	0.301	0.361	0.312
3w	0.201	0.228	0.260	0.315	0.387	0.348	0.259	0.296	0.397
4w	0.203	0.154	0.274	0.430	0.235	0.347	0.193	0.362	0.345
5w	0.254	0.227	0.214	0.452	0.228	0.517	0.257	0.358	0.434
6w	0.172	0.241	0.289	0.365	0.280	0.306	0.139	0.381	0.375
7w	0.234	0.221	0.182	0.482	0.228	0.403	0.253	0.336	0.338
8w	0.234	0.301	0.293	0.445	0.208	0.343	0.221	0.369	0.329
9w	0.269	0.234	0.253	0.455	0.318	0.397	0.149	0.355	0.436
10w	0.261	0.199	0.261	0.443	0.293	0.330	0.307	0.417	0.308

Control					
	SBP	SBP	SBP	SBP	SBP
1	107.2	99.6	99.0	98.5	99.0
2	105.3	101.1	94.9	100.3	99.8
3	107.0	97.7	99.7	99.6	96.9
4	104.3	98.8	102.4	100.2	100.7
5	104.7	98.3	101.6	101.4	99.6
	HR	HR	HR	HR	HR
1	303.0	289.0	283.0	281.7	295.6
2	286.8	280.2	280.1	278.8	286.8
3	288.1	274.7	280.8	283.3	290.5
4	288.3	293.4	284.9	281.0	293.5
5	285.8	281.9	284.8	285.9	306.7
	MF	MF	MF	MF	MF
1	0.441	0.384	0.431	0.369	0.382
2	0.400	0.374	0.386	0.415	0.417
3	0.491	0.383	0.437	0.413	0.379
4	0.402	0.405	0.455	0.420	0.431
5	0.528	0.377	0.448	0.445	0.417

Data (Fig 5)

			1	2	3	4	5
Before cast	SBP	pre	106.5	102.3	101.0	104.8	108.2
		post	83.1	77.5	84.6	83.4	85.4
	HR	pre	321.5	298.6	291.3	284.5	293.1
		post	446.6	459.7	456.4	433.1	477.1
During cast	SBP	pre	116.9	114.6	122.6	132.5	125.0
		post	78.2	75.6	66.1	67.6	89.3
	HR	pre	341.1	343.6	321.0	320.4	337.0
		post	479.6	487.4	467.4	421.3	459.8
After cast	SBP	pre	94.7	88.3	94.5	93.1	94.0
		post	97.2	84.9	91.0	90.8	92.4
	HR	pre	264.5	252.8	252.1	269.3	267.2
		post	379.8	359.0	336.7	338.9	368.1

Data (Fig 6)

Before cast removal									
Time	SBP	SBP	SBP	SBP	SBP	SBP	SBP	SBP	SBP
−55	85.8	112.0	104.6	98.5	103.7	104.3	105.2	105.7	99.4
−50	114.5	109.8	103.4	109.0	103.1	101.5	100.1	101.2	97.9
−45	110.7	106.0	106.5	103.7	99.2	106.5	105.2	100.5	99.3
−40	107.4	103.1	105.0	98.5	98.0	100.5	100.1	101.5	99.1
−35	105.0	104.6	106.0	95.3	96.4	96.7	102.6	106.3	100.6
−30	99.0	102.5	110.0	91.5	95.5	97.5	103.7	107.9	98.0
−25	94.3	104.1	106.0	92.5	93.3	101.9	99.9	109.3	96.2
−20	92.6	103.4	106.4	93.5	97.3	99.3	98.9	105.3	97.7
−15	93.5	101.7	106.8	95.8	93.5	96.3	100.0	103.3	100.8
−10	95.0	103.3	103.5	95.6	98.9	99.0	101.0	102.3	98.2
−5	92.4	103.4	106.0	102.9	96.6	99.4	104.4	103.4	96.6
0	95.1	113.0	119.3	112.0	96.0	104.8	120.7	116.9	105.2
5	114.0	118.6	121.4	122.8	102.4	106.9	111.8	130.3	107.2
10	116.3	126.5	129.5	119.2	108.8	110.5	117.3	135.2	115.1
15	117.7	132.1	136.2	121.5	110.9	110.2	119.0	134.4	126.0
20	118.4	128.6	131.8	118.9	114.5	111.4	125.4	136.8	135.8
25	117.1	129.2	137.3	113.2	111.0	117.9	124.9	135.2	137.3
30	115.9	127.3	132.5	105.3	110.5	120.2	122.2	131.8	134.7
35	112.6	124.3	128.2	108.9	112.7	121.1	122.4	130.6	134.9
40	114.3	124.3	131.0	110.7	112.5	122.7	126.1	130.5	129.4
45	113.7	125.7	126.9	110.2	117.9	122.7	128.5	130.7	126.9
50	113.3	128.7	127.8	111.5	123.7	121.0	133.5	128.9	129.7
55	117.2	131.8	126.4	113.6	122.7	126.1	133.7	127.4	133.3
60	114.1	131.7	126.7	114.0	122.4	128.1	134.4	133.8	128.7
65	114.9	130.8	131.6	113.4	123.3	125.0	137.7	132.0	125.9
70	119.9	132.5	130.1	113.2	125.6	127.2	133.6	131.7	122.0
75	120.5	132.8	132.9	112.2	125.5	130.2	133.0	126.9	126.0
80	117.7	132.0	131.3	115.2	126.2	128.0	130.7	126.2	129.8
85	117.7	132.2	131.0	114.3	122.6	128.3	128.5	126.3	131.7
90	126.7	134.0	128.8	113.1	124.1	127.8	130.3	126.9	128.8
95	130.4	136.0	140.6	121.7	127.8	125.4	137.6	128.7	129.5
100	128.0	130.2	135.5	120.6	125.4	121.0	131.6	135.5	124.6
105	123.2	126.5	128.5	119.0	120.3	119.2	127.0	127.8	117.4
110	119.4	123.3	122.3	116.2	114.9	118.7	118.9	122.2	118.3
115	115.4	123.0	122.6	102.1	111.7	108.1	112.8	117.1	125.6
120	112.2	124.6	116.7	94.9	104.4	108.0	113.1	119.9	118.9
125	110.2	123.9	116.7	93.4	99.0	107.7	107.7	115.0	118.5
130	107.3	116.7	113.5	93.1	94.4	105.2	111.2	113.4	110.9
135	106.0	113.9	111.1	91.0	99.3	103.2	106.6	114.5	108.3
140	105.4	116.6	108.1	95.6	95.3	105.1	103.2	109.2	110.2
145	103.8	117.6	109.3	91.7	91.3	100.6	104.2	106.8	108.9
150	101.6	115.5	107.8	87.7	89.3	100.6	107.6	102.8	101.5
155	101.5	112.1	110.8	86.8	94.6	101.8	101.2	101.6	103.0
160	99.4	106.7	107.5	85.6	90.2	95.5	105.3	100.4	103.9
165	99.6	106.1	112.7	85.4	87.0	97.2	109.3	99.6	98.9
170	100.6	108.0	112.1	86.5	96.6	95.3	106.6	99.0	100.4

After cast removal									
Time	SBP	SBP	SBP	SBP	SBP	SBP	SBP	SBP	SBP
−55	90.3	98.5	91.9	90.8	109.5	92.2	93.0	94.6	103.0
−50	95.5	95.9	93.9	100.5	82.5	91.6	99.9	95.5	104.8
−45	86.0	100.2	100.5	88.5	82.6	85.7	94.0	91.9	94.0
−40	82.8	98.5	102.1	87.5	82.0	86.4	95.0	94.5	88.1
−35	85.3	96.0	104.0	85.7	81.6	87.1	102.0	94.4	89.3
−30	83.5	92.4	96.2	95.3	78.4	89.4	95.4	94.7	85.8
−25	83.1	94.8	98.8	94.3	78.6	84.5	94.7	92.6	86.6
−20	88.5	98.3	97.6	92.3	78.3	90.8	93.6	94.5	89.5
−15	87.4	95.0	95.3	90.1	79.0	92.6	98.4	96.1	91.8
−10	92.5	94.3	97.8	90.1	84.6	94.7	95.8	93.4	86.5
−5	88.9	93.8	92.2	87.0	90.8	89.5	92.8	91.0	88.5
0	92.1	102.4	102.9	103.1	88.5	111.7	105.6	104.1	100.5
5	108.6	114.2	121.0	104.4	111.6	121.8	104.1	121.7	117.1
10	107.1	118.8	123.4	98.3	106.0	122.0	107.9	120.8	130.8
15	108.5	115.4	119.4	106.4	114.9	118.6	124.4	121.1	135.0
20	100.1	114.3	125.9	113.0	111.9	114.9	129.1	120.5	132.9
25	109.9	124.6	129.1	115.0	107.6	120.4	116.0	122.6	131.8
30	112.1	117.5	122.1	110.5	116.0	123.4	117.9	119.5	133.0
35	112.3	121.3	125.2	109.7	114.2	118.9	116.4	118.1	125.8
40	115.2	140.1	132.8	113.0	117.2	127.3	126.2	125.8	134.0
45	116.5	136.4	130.4	111.7	131.6	118.2	122.4	122.2	130.4
50	117.1	131.0	141.5	111.2	137.0	122.3	124.0	123.6	124.0
55	122.1	128.5	149.0	115.3	132.1	124.8	128.9	126.8	123.4
60	120.2	126.5	146.4	112.3	132.8	131.1	129.8	123.1	123.0
65	119.6	126.1	132.3	118.5	129.9	126.0	135.3	122.3	125.6
70	120.3	135.6	135.7	123.2	132.3	128.6	138.6	130.8	132.2
75	126.4	129.3	130.1	117.9	132.8	126.2	136.7	128.4	133.2
80	119.9	132.4	124.5	117.9	127.6	128.8	135.4	133.0	133.3
85	133.6	129.3	122.8	117.0	125.4	126.1	134.9	131.5	128.8
90	129.7	128.2	128.0	112.3	121.8	134.6	131.0	130.2	129.3
95	120.0	131.2	133.1	110.1	127.0	138.0	131.9	131.2	136.1
100	124.8	131.5	136.3	114.0	123.3	129.7	134.0	132.2	132.2
105	114.0	121.3	136.3	109.7	118.5	124.2	128.8	128.1	127.6
110	120.1	111.2	122.3	108.3	114.2	119.0	127.1	117.2	126.8
115	109.6	106.4	110.4	126.1	113.0	125.0	113.5	112.4	115.2
120	96.7	101.8	107.7	124.0	101.7	110.1	115.8	116.0	102.4
125	95.2	103.5	104.6	116.0	88.2	111.3	113.1	110.1	95.6
130	90.9	105.1	103.3	116.2	96.0	102.8	106.4	109.4	91.9
135	103.6	104.7	101.8	120.5	100.5	97.5	103.7	110.1	90.6
140	87.6	108.3	99.3	106.8	85.6	93.5	107.1	99.9	90.9
145	90.4	111.9	96.6	91.1	81.3	91.5	100.5	102.4	98.3
150	104.6	99.2	98.0	87.6	78.6	89.5	98.0	96.9	94.1
155	85.7	96.9	118.0	90.8	76.7	87.0	102.8	96.2	94.3
160	83.4	93.9	113.1	101.8	80.9	95.3	99.7	95.4	95.4
165	81.4	101.5	118.2	91.0	79.4	91.1	99.0	94.3	94.5
170	77.2	99.7	110.5	96.9	79.8	90.5	96.8	92.8	84.0

Data (Fig 7)

	ΔSBP	ΔSBP	ΔSBP	ΔSBP	ΔSBP	ΔSBP
B	25.7	18.2	26.8	23.8	28.8	29.9
C	28.2	25.1	30.5	38.6	26.1	22.1
4d	31.3	32.8	25.0	30.5	35.9	34.9
1w	28.7	25.2	34.1	29.6	36.4	36.6
2w	29.2	31.5	33.4	35.9	38.3	40.7
3w	31.0	27.6	26.6	34.2	36.7	36.7
4w	31.9	35.3	41.0	30.6	36.7	35.0
5w	29.8	26.5	42.7	32.2	33.9	41.4
6w	28.0	22.8	35.3	31.3	37.4	39.5
7w	36.8	28.8	41.1	33.0	37.7	33.4
8w	30.3	23.7	39.4	35.2	38.2	42.1
9w	38.3	29.7	32.1	39.0	39.8	33.8
10w	32.3	21.9	41.2	37.6	32.3	34.9

	ΔHR	ΔHR	ΔHR	ΔHR	ΔHR	ΔHR
B	150.8	133.5	156.2	135.2	156.9	164.1
C	157.6	150.8	148.2	181.4	158.2	156.8
4d	151.8	151.1	126.2	169.2	163.1	174.2
1w	130.2	106.2	135.8	140.8	174.8	200.4
2w	150.7	121.2	148.6	167.5	167.9	190.1
3w	144.5	130.2	116.3	147.6	135.0	163.8
4w	143.0	136.5	132.5	130.6	132.7	166.3
5w	125.0	127.8	157.7	168.2	157.7	161.2
6w	112.0	107.9	149.3	148.2	182.2	166.8
7w	149.0	104.9	152.6	161.5	164.6	141.5
8w	134.2	110.6	162.1	164.7	142.3	149.3
9w	132.0	97.4	145.5	172.2	166.7	141.4
10w	130.4	102.3	151.5	197.1	118.5	149.2
