# Supplementary material for: Changes in cardiovascular parameters in rats exposed to chronic widespread mechanical allodynia induced by hind limb cast immobilization
Source: PLoS One. 2021 Jan 19;16(1):e0245544. doi: 10.1371/journal.pone.0245544 (PMC7815128; doi:10.1371/journal.pone.0245544)
Supplement: S1 Table — (PDF) [file pone.0245544.s003.pdf]

S1 Table. Responses of systolic arterial blood pressure to phentolamine at various ages in normal rats

Rat ID	Timing matched to CPCP	SBP (mmHg)		ΔSBP
		Before PHE	After PHE	
1	During cast	110.5	89.0	21.5
2	1 w	104.5	85.8	18.7
3	4 w	106.6	86.9	19.7
4	5 w	102.3	77.5	24.8
5	8 w	109.5	67.5	42.0

CPCP: chronic post-cast pain model, SBP: systolic arterial blood pressure, PHE: phentolamine, w: weeks after cast removal
