# Supplementary material for: Changes in cardiovascular parameters in rats exposed to chronic widespread mechanical allodynia induced by hind limb cast immobilization
Source: PLoS One. 2021 Jan 19;16(1):e0245544. doi: 10.1371/journal.pone.0245544 (PMC7815128; doi:10.1371/journal.pone.0245544)
Supplement: S2 Table — (PDF) [file pone.0245544.s004.pdf]

S2 Table. Responses of systolic arterial blood pressure to double administration of phentolamine in normal rats.

Rat ID	1st PHE administration				2nd PHE administration			
	Timing matched to CPCP	SBP (mmHg)		ΔSBP	Timing matched to CPCP	SBP (mmHg)		ΔSBP
		Before PHE	After PHE			Before PHE	After PHE	
2	1 w	104.5	85.8	18.7	8 w	102.9	86.5	16.4
3	4 w	106.6	86.9	19.7	13 w	107.2	81.5	25.7

CPCP, chronic post-cast pain model; SBP, systolic arterial blood pressure; PHE, phentolamine; w, weeks after cast removal
